# Database selection and data gathering methods in systematic reviews of qualitative research regarding diabetes mellitus - an explorative study

**DOI:** 10.1186/s12874-021-01281-2

**Published:** 2021-04-30

**Authors:** Tobias Justesen, Josefine Freyberg, Anders N. Ø. Schultz

**Affiliations:** 1 Research unit, Department of Internal Medicine, Hospital of Southern Jutland, Sonderborg, Denmark; 2 Institute of Regional Health Research, University of Southern Denmark, Sonderborg, Denmark

**Keywords:** Databases, Qualitative research, Systematic review, Literature search, Diabetes mellitus

## Abstract

**Background:**

Systematic reviews (SRs) are considered one of the most reliable types of studies in evidence-based medicine. SRs rely on a comprehensive and systematic data gathering, including the search of academic literature databases. This study aimed to investigate which combination of databases would result in the highest overall recall rate of references when conducting SRs of qualitative research regarding diabetes mellitus. Furthermore, we aimed to investigate the current use of databases and other sources for data collection.

**Methods:**

Twenty-six SRs (published between 2010 and 2020) of qualitative research regarding diabetes mellitus, located through PubMed, met the inclusion criteria. References of the SRs were systematically hand searched in the six academic literature databases CINAHL, MEDLINE/PubMed, PsycINFO, Embase, Web of Science, and Scopus and the academic search engine Google Scholar. Recall rates were calculated using the total number of included references retrieved by the database or database combination divided by the total number of included references, given in percentage.

**Results:**

The SRs searched five databases on average (range two to nine). MEDLINE/PubMed was the most commonly searched database (100% of SRs). In addition to academic databases, 18 of the 26 (69%) SRs hand searched the reference lists of included articles. This technique resulted in a median (IQR) of 2.5 (one to six) more references being included per SR than by database searches alone. 27 (5.4%) references were found only in one of six databases (when Google Scholar was excluded), with CINAHL retrieving the highest number of unique references (*n* = 15). The combinations of MEDLINE/PubMed and CINAHL (96.4%) and MEDLINE/PubMed, CINAHL, and Embase (98.8%) yielded the highest overall recall rates, with Google Scholar excluded.

**Conclusions:**

We found that the combinations of MEDLINE/PubMed and CINAHL and MEDLINE/PubMed, CINAHL, and Embase yielded the highest overall recall rates of references included in SRs of qualitative research regarding diabetes mellitus. However, other combinations of databases yielded corresponding recall rates and are expected to perform comparably. Google Scholar can be a useful supplement to traditional scientific databases to ensure an optimal and comprehensive retrieval of relevant references.

## Background

Systematic reviews (SRs) are thorough reviews of the literature on a clearly outlined research question and are considered one of the most reliable types of studies in evidence-based medicine. Investigators are advised to search multiple academic databases and reference lists when conducting SRs [1, 2]. The Cochrane Handbook recommends searching at least Cochrane Central, MEDLINE and Embase as well as applying the MEDLINE search strategy, which should include a) a term for the health condition of interest, b) the intervention for evaluation, and c) the study design when conducting SRs of randomized controlled trials (RCTs) [2]. For qualitative evidence synthesis, Cochrane suggests using purposive sampling instead of the exhaustive approaches for quantitative research and recommends placing extra emphasis on searching for grey literature and in local databases [2]. An alternative to the traditional academic literature databases are academic search engines such as Google Scholar and Microsoft Academic Search. These search engines are free of charge and “crawl” the internet for relevant academic literature rather than search peer-reviewed published literature within a database. As a result, academic search engines can also find grey literature (documents not published by commercial publishers) such as academic theses and organization reports, reducing possible publication bias in a SR. [3] The process of searching through multiple academic databases and search engines can be tedious, as each has its own interface and requires separate search strings. For example, Boolean operators, phrase searching, truncation and use of parentheses can all differ between databases [4]. Therefore, to improve search quality, an information specialist’s involvement is generally recommended [2, 5, 6]. Investigators are naturally very interested in how many databases are necessary to achieve a suitable number of references when conducting a SR. However, it is equally important to know which databases will give the broadest search results and highest likelihood of unique references i.e. references not found elsewhere within a given field. These questions have been investigated earlier in qualitative research in general terms [7], within the field of depression [8] as well as in quantitative research [9–15] with one study exploring diabetes mellitus [16]. However, no previous studies have investigated SRs of qualitative research regarding diabetes mellitus. Diabetes mellitus is one of the most frequent chronic diseases in the twenty-first century, with a global prevalence estimated at 463 million people (9.3%) in 2019 and an estimated increase to 700 million (10.9%) by 2045 [17]. It is a disease that demands rigorous and comprehensive care, as patients need to control diet, exercise, medication and health check-ups with podiatrists, ophthalmologists and general practitioners or endocrinologists. At the same time, the patients do not necessarily sense the symptoms of the disease. Therefore, compliance is a substantial problem for this patient group [18], resulting in high occurrences of complications. It is essential to understand the barriers concerning the patients’ compliance. Unlike quantitative studies, qualitative studies offer an opportunity to understand the clinicians’, caregivers’, relatives’ and, most importantly, the patients’ point of view. In the field of diabetes mellitus, qualitative studies give insight into measures successful in maintaining compliance and impacting the lives of patients. This study aimed to investigate which combination of academic literature databases and academic search engines would result in the highest recall rate of references, when conducting SRs of qualitative research regarding diabetes mellitus. Furthermore, we aimed to investigate the current use of academic literature databases and search engines (hereafter jointly referred to as databases), information specialists and additional data gathering methods.

## Methods

### Inclusion and exclusion of SRs

SRs of qualitative research regarding diabetes mellitus were retrieved from the PubMed database for all entries before the day of inclusion (January 25, 2021). The search terms (“Qualitative Research” [MeSH]) and (“Diabetes Mellitus” [MeSH]) were combined using the Boolan operator “AND”, and the filter “Systematic reviews” was applied. Despite not applying language restrictions, the search only yielded English results. Likewise, no restriction to the year of publication was applied. SRs were systematically full text evaluated according to the inclusion and exclusion criteria. Inclusion criteria were SRs of either qualitative or mixed methods (both qualitative and quantitative) research regarding all subtypes of diabetes mellitus. Exclusion criteria were a) lack of a full list of databases searched for data collection in the SR, b) included references not extractable through the reference list or supplementary data, c) SRs which focused solely on other diseases than diabetes mellitus or d) SRs only quantitative in nature. Details about collected variables from each SR and the references are summarized in Table 1.

**Table 1:** Collected variables for the included SRs and references

Collected variables for SRs	
▪ Number of searched databases	
▪ Names of searched databases	
▪ Use of an information specialist	
▪ Search of reference lists of included studies	
▪ Use of additional data sources e.g. hand searched journals, key authors	
Collected variables for references	
▪ Recall in searched databases	

### Inclusion and exclusion of references from SRs

**Fig. 1:**
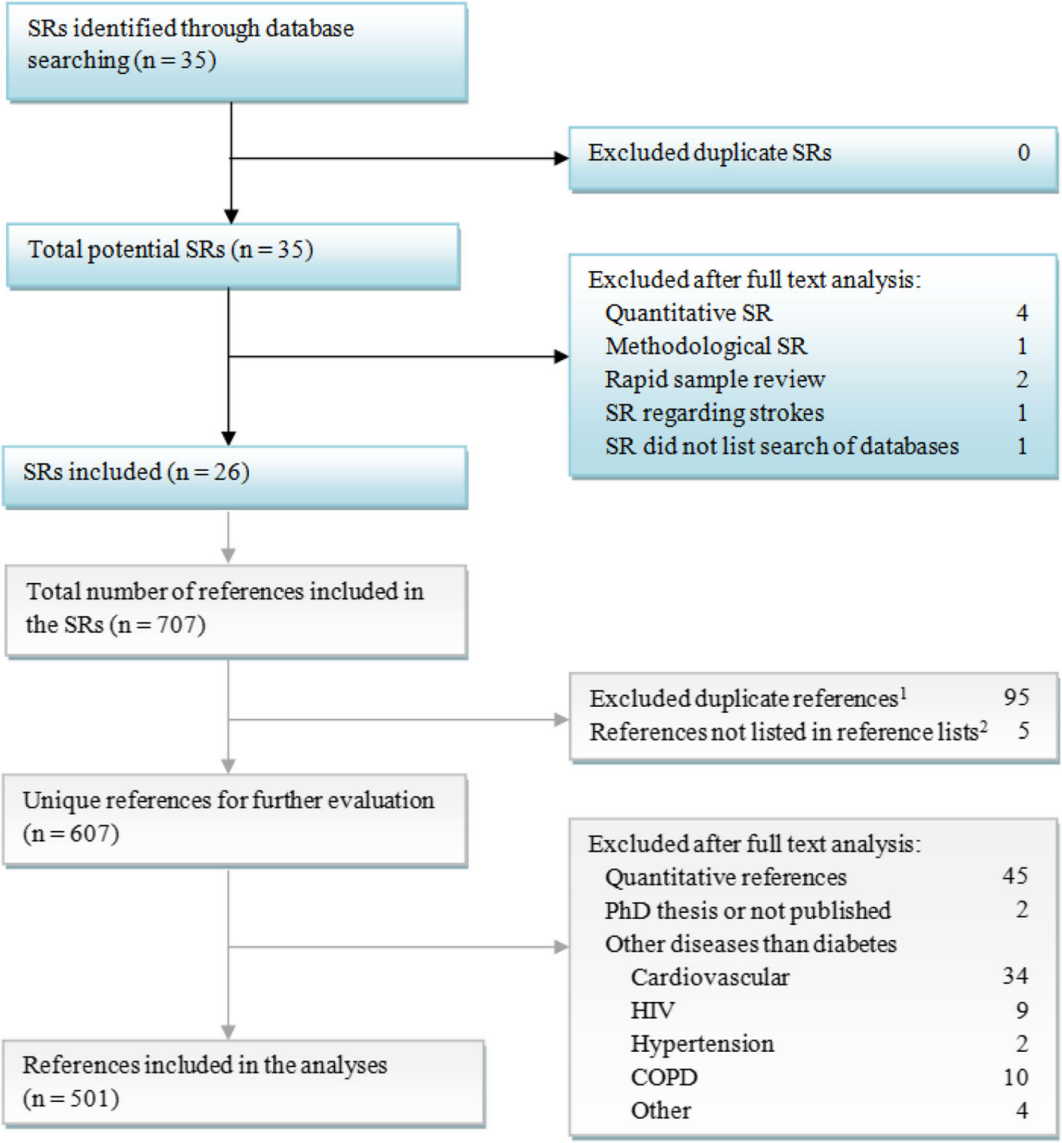
Flow diagram of the data collection process. ^1^ References included from more than one SR. ^2^ Two SRs [19, 20] included 85 references in total, but listed only 80 references in the reference lists. COPD = Chronic Obstructive Pulmonary Disorders. HIV = Human Immunodeficiency Virus

A list of all included references was extracted from each SR. Each reference was evaluated on whether it met the inclusion and exclusion criteria. Inclusion criterion was references included in one of the included SRs. Exclusion criteria were a) quantitative references included in mixed methods SRs, b) references of diseases other than diabetes mellitus included in SRs of multiple diseases and c) unpublished references. Figure 1 illustrates the inclusion process of the SRs and their references. All references were systematically hand searched in seven databases CINAHL, MEDLINE/PubMed, PsycINFO, Embase, Web of Science, Scopus, and Google Scholar. Social Science Citation Index (SSCI) was not investigated in this study, as it is one of six databases already included in Web of Science. MEDLINE, and PubMed were treated as one database because PubMed includes all MEDLINE references [21]. The references were initially searched by title. If the title search did not retrieve the reference, further searches, initially using the basic search functions and later using keywords, authors, and journals, were completed. For each reference, it was documented whether the reference was found and in which of the databases.

### Statistical analyses

The number and frequency of databases searched were described in absolute numbers and mean, median, and interquartile range (IQR). Calculations for correlation of the number of databases and year published were performed using Poisson regression. Searches of reference lists were described in absolute numbers as well as median and range. The contribution of references from each individual database and the various combinations of their combined contribution were calculated as absolute numbers and recall or combined recall. Recall rates were calculated using the total number of included references retrieved by the database or database combination divided by the total number of included references, given in percentage. All calculations of recall rates and unique number of references per database were performed firstly with all seven databases and secondly with Google Scholar excluded, since Google Scholar’s precision in structured literature searches has been reported to be low despite high recall rates (the topic of which is further addressed in the Discussion section) [22, 23]. All statistical analyses were performed using RStudio for Windows (v. 4.0.2 RStudio v. 1.3.1093).

## Results

### Inclusion and exclusion of SRs and references

The initial search of PubMed, with the search syntax defined in the methods section, yielded a result of 35 SRs. Nine SRs were excluded, the process of which is detailed in Fig. 1. A total of 26 SRs met the inclusion criteria and were included in the study. All SRs included were published between 2010 and 2020. No correlations were found between the year of publication, and the number of databases searched. See Appendix 1 for an overview of included SRs. The 26 SRs contained a total of 707 references. Five references could not be extracted as two SRs included 85 references in total, but only listed 80 references in the reference lists. Two hundred one references were excluded (see Fig. 1 for further details), and 501 unique, qualitative studies concerning diabetes mellitus were included. A median (IQR) of 12.5 (6 to 24) references were included per SR.

### Databases and their frequency of use

**Fig. 2:**
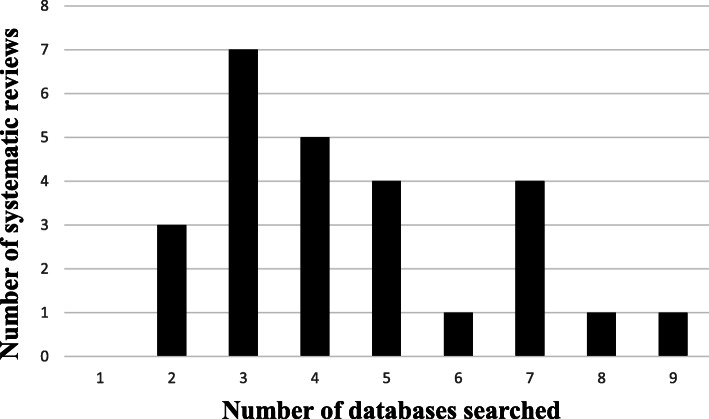
Number of databases searched by SRs of qualitative research regarding diabetes mellitus

The mean and median number of databases searched by the SRs were five and four, respectively, with a range from two to nine databases (Fig. 2).

**Fig. 3:**
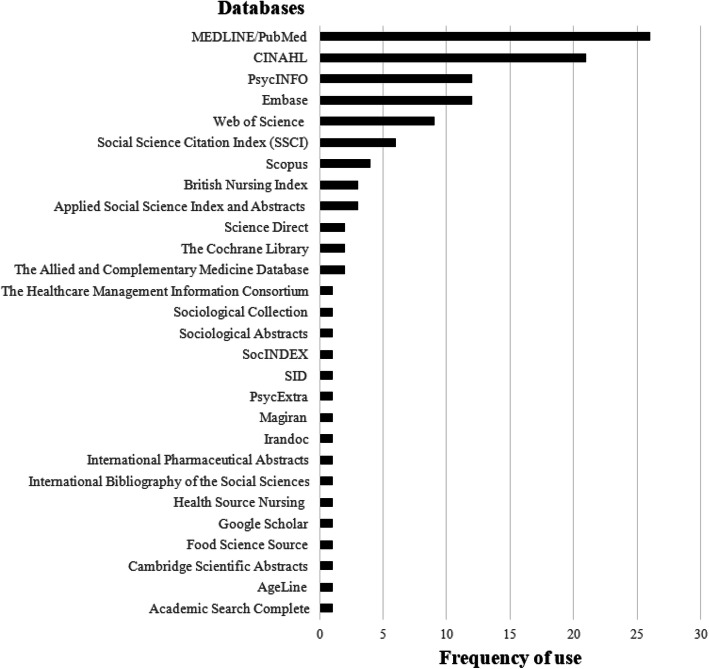
Frequency of database use by the included 26 SRs

The 26 SRs searched 28 different databases, of which 12 were reported more than once. MEDLINE/PubMed was the most searched database applied by all SRs (100%), followed by CINAHL, which was searched by 21 out of the 26 SRs (81%). Embase and PsycINFO were the third and fourth most searched databases, both searched by 12 SRs (46%) (Fig. 3).

### The use of information specialists and additional sources

Only one (4%) SR [24] involved an information specialist when choosing databases. Another SR [25] used a search filter developed by an information specialist, while the remaining 24 SRs did not mention using an information specialist. Eighteen of the 26 (69%) SRs searched reference lists of included articles (two of the 18 SRs did not present the results of these searches). This resulted in a median (IQR) of 2.5 (one to six) more references being included per SR than by database searches alone. In total, the 16 SRs included 48 references from searching reference lists of included articles. These 48 references are included in the total number of 501 references. Three SRs exclusively searched databases, while the remaining SRs, in addition to databases, hand searched journals, key authors, and other sources.

### Unique references per database

The seven databases MEDLINE /PubMed, CINAHL, Embase, PsycINFO, Web of Science, Scopus, and Google Scholar were investigated individually. A total of 9 (1.8%) references were unique to only one of these seven databases. Table 2 shows the number of unique references for each database. Embase retrieved the highest number of unique references followed by CINAHL, Google Scholar and MEDLINE/PubMed. The databases were also investigated excluding Google Scholar, and in this case, CINAHL retrieved the highest number of unique references (*n* = 15), followed by Embase (*n* = 5), and MEDLINE/PubMed (*n* = 5).

**Table 2:** Number of unique references in each database

Databases	No. of SRs that searched the database	No. of SRs with unique references	No. of unique references in the database	No. of SRs with unique references - GSc excluded	No. of unique references in the database – GSc excluded
MEDLINE/PubMed	26	1 (4%)	1 (11%)	5 (19%)	5 (19%)
CINAHL	20	2 (10%)	2 (22%)	7 (35%)	15 (56%)
Embase	12	1 (8%)	4 (44%)	1 (8%)	5 (19%)
PsycINFO	12	0 (0%)	0	0 (0%)	0 (0%)
Web of Science	8	0 (0%)	0	1 (13%)	1 (4%)
Scopus	4	0 (0%)	0	1 (25%)	1 (4%)
Google Scholar	2	2 (100%)	2 (22%)	–	–

*No*. number, *GSc* Google Scholar

### Search of databases and their overall recall

For each database and their combinations, the recall rates of the 501 individual references were calculated. The calculations are shown in Table 3. Google Scholar showed the highest overall recall rate (97%), and Scopus, the second-highest overall recall rate (92%). The seven databases had overall recall rates between 39 to 97%. The combination of Google Scholar and Embase retrieved the highest overall recall rate (99%) regarding combinations of two databases. Excluding Google Scholar, the combination of two databases with the highest overall recall rate was MEDLINE/PubMed and CINAHL with 96%. The combination of three databases with the highest overall recall rate was Google Scholar, Embase, and either MEDLINE/PubMed (99.6%) or CINAHL (99.6%). Excluding Google Scholar, the combination of three databases with the highest overall recall rate was MEDLINE/PubMed, Embase, and CINAHL (98.8%).

**Table 3:** Individual and combined recall rates of references in the included databases

Databases	References found (n)	Overall recall^**a**^ (%)	Median recall^**b**^ (%)	Minimum recall^**c**^ (%)	100% recall^**d**^ (%)	Number of SRs that searched the database, n (% of the 26 SRs)
GSc	486	97.0	100.0	84.6	73.1	1 (3.9)
SCO	462	92.2	76.8	50.0	38.5	4 (15.4)
EMB	443	88.4	89.3	60.0	23.1	12 (46.2)
ML/PM	436	87.0	93.3	62.5	42.3	26 (100.0)
CIN	386	77.1	80.0	25.0	11.5	20 (76.9)
WoS	386	77.1	76.8	50.0	11.5	8 (30.8)
PSI	195	38.9	40.0	0.0	0.0	12 (46.2)
**Combinations of two databases**^e^						
GSc + EMB	497	99.2	100.0	87.5	84.6	1 (3.8)
GSc + SCO	492	98.2	100.0	84.6	88.5	0 (0)
GSc + Wos	491	98.0	100.0	87.5	76.9	0 (0)
ML/PM + CIN	483	96.4	100.0	75.0	69.2	20 (76.9)
CIN + SCO	482	96.2	100.0	77.8	65.4	3 (11.5)
EMB + CIN	481	96.0	100.0	75.0	65.4	9 (34.6)
**Combinations of three databases**^f^						
GSc + ML/PM + EMB	499	99.6	100.0	87.5	92.3	1 (3.8)
GSc + EMB + CIN	499	99.6	100.0	92.3	92.3	1 (3.8)
GSc + EMB + SCO	498	99.4	100.0	87.5	88.5	0 (0)
ML/PM + EMB + CIN	495	98.8	100.0	80.0	80.8	9 (34.6)
EMB + CIN + WoS	493	98.4	100.0	83.3	76.9	1 (3.8)
ML/PM + CIN + WoS	492	98.2	100.0	83.3	80.8	6 (23.1)

*CIN* CINAHL, *EMB* embase, *GSc* Google Scholar, *ML/PM* MEDLINE/PubMed, *PSI* PsycINFO, *SCO* Scopus, *WoS* Web of Science

^a^Overall recall: The total number of included references retrieved by the databases divided by the total number of included references

^b^Median recall: The median value of recall per systematic review

^c^Minimum recall: The lowest value of recall per systematic review

^d^100% recall: The percentage of systematic reviews for which the database or database combination retrieved all included references

^e^Results of the combination of two databases are presented for the three combinations that yielded the highest results both with and without Google Scholar. For the results of the remaining database combinations see Appendix 2

^f^Results of the combination of three databases are presented for the three combinations that yielded the highest results both with and without Google Scholar. For the results of the remaining database combinations see Appendix 2

## Discussion

Our study underlines the importance of choosing the optimal combination of databases when conducting a qualitative SR regarding diabetes mellitus. It has previously been suggested that a SR must include at least 95% of the publications on any given subject to be acceptable [9]. We found that the combinations of MEDLINE/PubMed and CINAHL (96.4%) and MEDLINE/PubMed, CINAHL, and Embase (98.8%) yielded the highest overall recall rates (when combining two and three databases, respectively), with Google Scholar excluded from the analyses. However, other combinations of databases yielded corresponding recall rates and are expected to perform comparably. Furthermore, CINAHL retrieved the highest number of unique references (*n* = 15), followed by MEDLINE/PubMed (*n* = 5), and Embase (n = 5), when Google Scholar was excluded. Based on these findings, we recommend searching at least the combination of MEDLINE/PubMed and CINAHL, when conducting qualitative SRs regarding diabetes mellitus (applied by 20 of 26 SRs).

These results contrast a previous study concluding that the combination of Scopus, CINAHL and ProQuest Dissertations and Thesis Global (hereafter referred to as ProQuest) contributed to the highest number of unique references for qualitative SRs [7]. ProQuest was not investigated in this study, as unpublished references were excluded. However, this only comprised two references and ProQuest would therefore not be expected to retrieve a high number of unique references. In alignment with previous findings [7, 26], our data showed that CINAHL retrieved the highest number of unique references (excluding Google Scholar), therefore suggesting CINAHL to be highly relevant when searching literature for qualitative diabetes mellitus research. CINAHL focuses on nursing and allied health research, a content that may be too narrow when researching multi- or interdisciplinary health science literature. In these cases, multidisciplinary databases such as Scopus and Web of Science could prove higher yielding [27]. Therefore, the nature of the research questions should be carefully considered when deciding the optimum combination of databases.

Google Scholar had the highest individual overall recall rate in this study (97%) and adds further value with the identification of grey literature [3]. However, databases such as ProQuest and GreySource offer similar access to grey literature. Despite the advantages of Google Scholar, its precision in structured literature searches has previously reported to be low [22, 23]. Google Scholar has many significant limitations, including search expressions being limited to 256 characters, displaying a maximum of 1000 results of the complete results without explaining how the order of results has been made and no bulk export options. Therefore, this search engine has previously been assessed as inadequate as a standalone resource for data gathering, when conducting comprehensive search activities, such as SRs [3]. We recommend Google Scholar be used as a supplement to the traditional scientific database searches in order to enhance retrieval of unique or unpublished references.

The majority of SRs searched reference lists of the included articles, similar to previous findings [28]. It can be argued that a comprehensive search in the optimal combination of databases would render the search of reference lists redundant, which our data on overall recall rates supports. However, whether a reference is present in a database does not directly translate into it being found with a given search string. In conclusion, searching reference lists is a valid way of searching for additional references not found by database searches alone. Two of the 26 SRs used either an information specialist or a search filter developed by an information specialist. These results contradicts a prior quantitative study that reported 51% of SRs used a librarian, though only 64% of these SRs actually reported this use [5]. Although, our findings are insufficient in making recommendations, we recommend consulting an information specialist before conducting database searches due to the challenge of each database requiring different search strings.

MEDLINE and PubMed were treated as one database due to the major overlap of references to avoid misleading results. However, it might be relevant to treat them as independent databases when conducting academic literature searches. PubMed includes all MEDLINE references as well as up-to-date citations, books and book chapters, and references from journals not indexed in MEDLINE, such as PMC journals [14, 29]. The larger quantity of content in PubMed compared to MEDLINE (91% of PubMed content is indexed in MEDLINE [21],) might contribute to more relevant references when conducting a SR. On the other hand, PMC literature has been criticized for potentially reducing the quality of PubMed, due to its informal reevaluation process (prior to 2017), though most manuscripts in PMC are also published in MEDLINE indexed journals [21]. For these reasons, we recommend the use of PubMed over MEDLINE.

### Limitations

This study has several limitations. Firstly, the SRs included in this study were found through the database PubMed. Other databases were not searched for SRs of qualitative research regarding diabetes mellitus. Secondly, the search string solely used MeSH terms, and because of this, may not have recovered all qualitative SRs of diabetes mellitus in the PubMed database contributing to selection bias. However, as this is an exploratory study and not a SR or meta-analysis, a sample of collectable data was assessed to be sufficient. Thirdly, since we only investigated the topic of SRs of qualitative research regarding diabetes mellitus, our results may not apply to other diseases or topic of research. Fourthly, not all databases were investigates in this study such as SSCI and British Nursing Index, which were the sixth and eighth most frequently searched databases. It is possible that combinations including these databases may have resulted in different conclusions. Fifthly, whether a reference is present in a database does not directly translate into whether it would have been found using a given search string. Therefore, our results may not be directly transferable to the search of references when conducting a SR. Sixthly, the recall rates in this study were derived from the references included in the SRs and not from the actual number of references available and relevant for the same SRs at their time of inclusion. There were likely relevant references on qualitative research on diabetes mellitus not included in the SRs. These references, if included in our study, might alter the results and recommendations for database selection.

## Conclusions

We found that the combinations of MEDLINE/PubMed and CINAHL (96.4%) and MEDLINE/PubMed, CINAHL, and Embase (98.8%) yielded the highest overall recall rates (when Google Scholar was excluded from the analyses) of references included in SRs of qualitative research regarding diabetes mellitus. Other combinations of databases did, however, yield corresponding recall rates and are expected to perform comparably. Google Scholar can be a useful supplement to traditional scientific databases to ensure an optimal and comprehensive retrieval of relevant references, both academic and grey literature. Further research on the subject should try to establish whether our findings within the field of diabetes mellitus are similar to other disease areas within qualitative research.

## Data Availability

The datasets used and/or analyzed during the current study are available from the corresponding author on reasonable request.

## Appendix 1

**Table 4:** Overview of included SRs and references

Included SRs (*n* = 26)	Included number of references (*n* = 501)
Messina J, Campbell S, Morris R, Eyles E, Sanders C. A narrative systematic review of factors affecting diabetes prevention in primary care settings. PLoS One. 2017;12 (5).	15
Al Hamid A, Ghaleb M, Aljadhey H, Aslanpour Z. A systematic review of qualitative research on the contributory factors leading to medicine-related problems from the perspectives of adult patients with cardiovascular diseases and diabetes mellitus. BMJ Open. 2014;4 (9).	15
Majeed-Ariss R, Jackson C, Knapp P, Cheater FM. A systematic review of research into black and ethnic minority patients’ views on self-management of type 2 diabetes. Heal Expect. 2015;18 (5):625–42.	50
Rushforth B, McCrorie C, Glidewell L, Midgley E, Foy R. Barriers to effective management of type 2 diabetes in primary care: Qualitative systematic review. Br J Gen Pract. 2016;66 (643):e114–27.	33
Mohammad Mohseni, Tahereh Shams Ghoreishi, Sousan Houshmandi, Ahmad Moosavi, Saber Azami-Aghdash, Zoleykha Asgarlou. Challenges of managing diabetes in Iran: meta-synthesis of qualitative studies. BMC Health Services Research. 2020;20 (1).	12
Brundisini F, Giacomini M, DeJean D, Vanstone M, Winsor S, Smith A. Chronic disease patients’ experiences with accessing health care in rural and remote areas: A systematic review and qualitative meta-synthesis. Ont Health Technol Assess Ser. 2013;13 (15):1–33.	5
Van Ryswyk E, Middleton P, Hague W, Crowther C. Clinician views and knowledge regarding healthcare provision in the postpartum period for women with recent gestational diabetes: A systematic review of qualitative/survey studies. Diabetes Research and Clinical Practice. 2,014,106 (3):401–1.	13
Vanstone M, Rewegan A, Brundisini F, Giacomini M, Kandasamy S, Dejean D. Diet modification challenges faced by marginalized and nonmarginalized adults with type 2 diabetes: A systematic review and qualitative meta-synthesis. Chronic Illness. 2017;13 (1):217–35.	108
Joseph Ngmenesegre Suglo, Catrin Evans. Factors influencing self-management in relation to type 2 diabetes in Africa: A qualitative systematic review. PLoS ONE. 2020;15 (10).	16
Wilkinson A, Whitehead L, Ritchie L. Factors influencing the ability to self-manage diabetes for adults living with type 1 or 2 diabetes. International Journal of Nursing Studies. 2014;51 (1):111–22.	27
Campbell F, Lawton J, Rankin D, Clowes M, Coates E, Heller S, et al. Follow-Up Support for Effective type 1 Diabetes self-management (The FUSED Model): A systematic review and meta-ethnography of the barriers, facilitators and recommendations for sustaining self-management skills after attending a structured education programme. BMC Health Serv Res. 2018;18 (1).	17
Vanstone M, Giacomini M, Smith A, Brundisini F, DeJean D, Winsor S. How diet modification challenges are magnified in vulnerable or marginalized people with diabetes and heart disease: A systematic review and qualitative meta-synthesis. Ont Health Technol Assess Ser. 2013;13 (14):1–40.	5
Long H, Bartlett YK, Farmer AJ, French DP. Identifying brief message content for interventions delivered via mobile devices to improve medication adherence in people with type 2 diabetes mellitus: A rapid systematic review. Journal of Medical Internet Research. 2019;21 (1).	4
Walker RC, Tong A, Howard K, Palmer SC. Patient expectations and experiences of remote monitoring for chronic diseases: Systematic review and thematic synthesis of qualitative studies. Int J Med Inform. 2019;124:78–85.	4
DeJean D, Giacomini M, Vanstone M, Brundisini F. Patient experiences of depression and anxiety with chronic disease: A systematic review and qualitative meta-synthesis. Ont Health Technol Assess Ser. 2013;13 (16):1–33.	4
Vanstone M, Rewegan A, Brundisini F, Dejean D, Giacomini M. Patient perspectives on quality of life with uncontrolled type 1 diabetes mellitus: A systematic review and qualitative meta-synthesis. Ontario Health Technology Assessment Series. 2015;15 (17):1–29.	28
Jain SR, Sui Y, Ng CH, Chen ZX, Goh LH, Shorey S. Patients’ and healthcare professionals’ perspectives towards technology-assisted diabetes self-management education. A qualitative systematic review. 2020;15 (8).	15
Shaw RL, Holland C, Pattison HM, Cooke R. Patients’ perceptions and experiences of cardiovascular disease and diabetes prevention programmes: A systematic review and framework synthesis using the Theoretical Domains Framework. Social Science and Medicine. 2016;156:192–203.	6
Whittemore R, Jaser S, Chao A, Jang M, Grey M. Psychological Experience of Parents of Children With Type 1 Diabetes: A Systematic Mixed-Studies Review. Diabetes Educ. 2012;38 (4):562–79.	12
Spencer J, Cooper H, Milton B. Qualitative studies of type 1 diabetes in adolescence: A systematic literature review. Pediatric Diabetes. 2010;11 (5):364–75.	26
Zuniga JA, Wright C, Fordyce J, West Ohueri C, Garciá AA. Self-Management of HIV and Diabetes in African American Women: A Systematic Review of Qualitative Literature. Diabetes Educ. 2018;44 (5):419–34.	2
Jones E, Sinclair JMA, Holt RIG, Barnard KD. Social networking and understanding alcohol-associated risk for people with type 1 diabetes: Friend or foe? Diabetes Technol Ther. 2013;15 (4):308–14.	6
Due-Christensen M, Zoffmann V, Willaing I, Hopkins D, Forbes A. The Process of Adaptation Following a New Diagnosis of Type 1 Diabetes in Adulthood: A Meta-Synthesis. Qual Health Res. 2018;28 (2):245–58.	8
Saunders T. Type 2 diabetes self-management barriers in older adults: An integrative review of the qualitative literature. J Gerontol Nurs. 2019;45 (3):43–54.	9
Villalba C, Jaiprakash A, Donovan J, Roberts J, Crawford R. Unlocking the Value of Literature in Health Co-Design: Transforming Patient Experience Publications into a Creative and Accessible Card Tool. Patient. 2018;11 (6):637–48.	12
Van Ryswyk E, Middleton P, Shute E, Hague W, Crowther C. Women’s views and knowledge regarding healthcare seeking for gestational diabetes in the postpartum period: A systematic review of qualitative/survey studies. Diabetes Research and Clinical Practice. 2015;110 (2):109–22.	49

## Appendix 2

**Table 5:** Databases included and their individual and combined recall of references

Combinations of two databases	References found (n)	Overall recall^**a**^ (%)	Median recall^**b**^ (%)	Minimum recall^**c**^ (%)	100.0% recall^**d**^ (%)	Number of SRs that searched the database. n (% of the 26 SRs)
GSc + EMB	497	99.2	100.0	87.5	84.6	1 (3.8)
GSc + SCO	492	98.2	100.0	84.6	88.5	0 (0)
GSc + WoS	491	98.0	100.0	87.5	76.9	0 (0)
GSc + ML/PM	490	97.8	100.0	85.7	80.8	1 (3.8)
GSc + CIN	490	97.8	100.0	84.6	76.9	1 (3.8)
GSc + PSI	487	97.2	100.0	84.6	73.1	1 (3.8)
ML/PM + CIN	483	96.4	100.0	75.0	69.2	20 (76.9)
CIN + SCO	482	96.2	100.0	77.8	65.4	3 (11.5)
EMB + CIN	481	96.0	100.0	75.0	65.4	9 (34.6)
EMB + SCO	477	95.2	98.6	75.0	50.0	1 (3.8)
ML/PM + SCO	475	94.8	100.0	75.0	57.7	4 (15.4)
WoS + SCO	470	93.8	95.1	75.0	38.5	2 (7.7)
ML/PM + EMB	467	93.2	95.5	62.5	46.2	12 (46.2)
EMB + WoS	464	92.6	92.8	62.5	34.6	6 (23.1)
PSI + SCO	464	92.6	95.1	75.0	38.5	3 (11.5)
CIN + WoS	461	92.2	93.2	58.3	34.6	6 (23.1)
ML/PM + WoS	455	90.8	100.0	62.5	53.9	8 (30.8)
EMB + PSI	455	90.8	91.9	62.5	26.9	7 (26.9)
ML/PM + PSI	445	88.8	96.7	62.5	50.0	12 (46.2)
CIN + PSI	411	82.0	83.3	25.0	19.2	10 (38.5)
PSI + WoS	409	81.6	83.3	50.0	19.2	4 (15.4)
**Combinations of three databases**						
GSc + ML/PM + EMB	499	99.6	100.0	87.5	92.3	1 (3.8)
GSc + EMB + CIN	499	99.6	100.0	92.3	92.3	1 (3.8)
GSc + EMB + SCO	498	99.4	100.0	87.5	88.5	0 (0)
GSc + EMB + PSI	497	99.2	100.0	87.5	84.6	1 (3.8)
GSc + EMB + WoS	497	99.2	100.0	87.5	84.6	0 (0)
ML/PM + EMB + CIN	495	98.8	100.0	80.0	80.8	9 (34.6)
GSc + ML/PM + CIN	494	98.6	100.0	89.8	88.5	1 (3.8)
GSc + CIN + WoS	494	98.6	100.0	91.8	84.6	0 (0)
GSc + CIN + SCO	494	98.6	100.0	84.6	92.3	0 (0)
GSc + WoS + SCO	494	98.6	100.0	87.5	88.5	0 (0)
GSc + ML/PM + WoS	493	98.4	100.0	87.5	84.6	0 (0)
GSc + ML/PM + SCO	493	98.4	100.0	87.5	88.5	0 (0)
EMB + CIN + WoS	493	98.4	100.0	83.3	76.9	1 (3.8)
ML/PM + CIN + WoS	492	98.2	100.0	83.3	80.8	6 (23.1)
GSc + PSI + SCO	492	98.2	100.0	84.6	88.5	0 (0)
GSc + CIN + PSI	491	98.0	100.0	84.6	80.8	1 (3.8)
GSc + PSI + WoS	491	98.0	100.0	87.5	76.9	0 (0)
ML/PM + CIN + SCO	490	97.8	100.0	85.7	80.8	3 (11.5)
GSc + ML/PM + PSI	490	97.8	100.0	85.7	80.8	1 (3.8)
EMB + CIN + SCO	489	97.6	100.0	75.0	73.1	5 (19.2)
CIN + WoS + SCO	487	97.21	100.0	77.8	65.4	1 (3.8)
ML/PM + CIN + PSI	486	97.0	100.0	75.0	76.9	10 (38.5)
ML/PM + EMB + SCO	483	96.4	100.0	75.0	61.5	1 (3.8)
EMB + CIN + PSI	483	96.4	100.0	75.0	69.2	6 (23.1)
CIN + PSI + SCO	483	96.4	100.0	77.8	65.4	3 (11.5)
ML/PM + WoS + SCO	480	95.8	100.0	75.0	65.4	2 (7.7)
EMB + PSI + SCO	478	95.4	98.6	75.0	50.9	1 (3.8)
EMB + WoS + SCO	478	95.4	98.6	75.0	50.0	0 (0)
ML/PM + EMB + WoS	476	95.0	100.0	62.5	57.7	6 (23.1)
ML/PM + PSI + SCO	476	95.0	100.0	75.0	57.7	3 (11.5)
ML/PM + EMB + PSI	474	94.6	100.0	62.5	53.9	7 (26.9)
PSI + WoS+ SCO	471	94.0	95.1	75.0	38.5	1 (3.8)
EMB + PSI + WoS	468	93.4	94.0	62.5	38.5	3 (11.5)
CIN + PSI + WoS	466	93.0	95.7	58.3	42.3	3 (11.5)
ML/PM + PSI + WoS	459	91.6	100.0	62.5	53.9	4 (15.4)

*CIN* CINAHL, *EMB* Embase, *GSc* Google Scholar, *ML/PM* MEDLINE/PubMed, *PSI* PsycINFO, *SCO* SCO, *WoS* Web of Science

^a^Overall recall: The total number of included references retrieved by the databases divided by the total number of included references retrieved by all databases

^b^Median recall: The median value of recall per systematic review

^c^Minimum recall: The lowest value of recall per systematic review

^d^100.0% recall: The percentage of reviews for which the database or database combination retrieved all included references

## Abbreviations

CINAHL: Cumulative Index to Nursing and Allied Health Literature
CIN: CINAHL
COPD: Chronic Obstructive Pulmonary Disorders
EMB: Embase
GSc: Google Scholar
HIV: Human Immunodeficiency Virus
IQR: Interquartile Range
ML/PM: MEDLINE/PubMed
PhD: Philosophiae Doctor
PSI: PsycINFO
RCT: Randomized controlled trial
SCO: Scopus
SR: Systematic review
SSCI: Social sciences citation index
WoS: Web of science

## References

[1] Muka T, Glisic M, Milic J, Verhoog S, Bohlius J, Bramer W, Chowdhury R, Franco OH (2020). A 24-step guide on how to design, conduct, and successfully publish a systematic review and meta-analysis in medical research. Eur J Epidemiol.

[2] Higgins JPT, Thomas J, Chandler J, Cumpston M, Li T, Page MJ, Welch VA (editors). Cochrane Handbook for Systematic Reviews of Interventions version 6.2 (updated February 2021). Cochrane. 2021. Available from www.training.cochrane.org/handbook.

[3] Haddaway NR, Collins AM, Coughlin D, Kirk S (2015). The role of google scholar in evidence reviews and its applicability to grey literature searching. Plos One.

[4] Bramer WM, de Jonge GB, Rethlefsen ML, Mast F, Kleijnen J (2018). A systematic approach to searching: an efficient and complete method to develop literature searches. J Med Libr Assoc.

[5] Koffel JB. Use of recommended search strategies in systematic reviews and the impact of librarian involvement: a cross-sectional survey of recent authors. PLoS One. 2015;10(5):e0125931. 10.1371/journal.pone.0125931 PMC4418838 25938454

[6] Rethlefsen ML, Farrell AM, Osterhaus Trzasko LC, Brigham TJ (2015). Librarian co-authors correlated with higher quality reported search strategies in general internal medicine systematic reviews. J Clin Epidemiol.

[7] Frandsen TF, Gildberg FA, Tingleff EB (2019). Searching for qualitative health research required several databases and alternative search strategies: a study of coverage in bibliographic databases. J Clin Epidemiol.

[8] Wright JM, Cottrell DJ, Mir G (2014). Searching for religion and mental health studies required health, social science, and grey literature databases. J Clin Epidemiol.

[9] Bramer WM, Rethlefsen ML, Kleijnen J, Franco OH (2017). Optimal database combinations for literature searches in systematic reviews: a prospective exploratory study. Syst Rev.

[10] Hartling L, Featherstone R, Nuspl M, Shave K, Dryden DM, Vandermeer B (2016). The contribution of databases to the results of systematic reviews: a cross-sectional study. BMC Med Res Methodol.

[11] Vassar M, Yerokhin V, Sinnett PM, Weiher M, Muckelrath H, Carr B, Varney L, Cook G (2017). Database selection in systematic reviews: an insight through clinical neurology. Health Inf Libr J.

[12] Halladay CW, Trikalinos TA, Schmid IT, Schmid CH, Dahabreh IJ (2015). Using data sources beyond PubMed has a modest impact on the results of systematic reviews of therapeutic interventions. J Clin Epidemiol.

[13] Aagaard T, Lund H, Juhl C (2016). Optimizing literature search in systematic reviews - are MEDLINE, EMBASE and CENTRAL enough for identifying effect studies within the area of musculoskeletal disorders?. BMC Med Res Methodol.

[14] Frandsen TF, Eriksen MB, Hammer DMG, Christensen JB (2019). PubMed coverage varied across specialties and over time: a large-scale study of included studies in Cochrane reviews. J Clin Epidemiol.

[15] Frandsen TF, Brandt M, Mortan D, Hammer G, Buck J, Albert J (2021). Using Embase as a supplement to PubMed in Cochrane reviews differed across fields. J Clin Epidemiol.

[16] Royle P, Bain L, Waugh N (2005). Systematic reviews of epidemiology in diabetes: finding the evidence. BMC Med Res Methodol.

[17] Saeedi P, Petersohn I, Salpea P, Malanda B, Karuranga S, Unwin N, et al. Global and regional diabetes prevalence estimates for 2019 and projections for 2030 and 2045: Results from the International Diabetes Federation Diabetes Atlas, 9(th) edition. Diabetes Res Clin Pract. 2019;157:107843. 10.1016/j.diabres.2019.107843 31518657

[18] Cramer JA (2004). A systematic review of adherence with medications for diabetes. Diabetes Care.

[19] Vanstone M, Giacomini M, Smith A, Brundisini F, DeJean D, Winsor S (2013). How diet modification challenges are magnified in vulnerable or marginalized people with diabetes and heart disease: a systematic review and qualitative meta-synthesis. Ont Health Technol Assess Ser.

[20] DeJean D, Giacomini M, Vanstone M, Brundisini F (2013). Patient experiences of depression and anxiety with chronic disease: a systematic review and qualitative meta-synthesis. Ont Health Technol Assess Ser..

[21] Williamson PO, Minter CIJ (2019). Exploring PubMed as a reliable resource for scholarly communications services. J Med Libr Assoc..

[22] Boeker M, Vach W, Motschall E. Google Scholar as replacement for systematic literature searches: good relative recall and precision are not enough. BMC Med Res Methodol. 2013;13:131. 10.1186/1471-2288-13-131 PMC3840556 24160679

[23] Shultz M (2007). Comparing test searches in PubMed and Google scholar. J Med Libr Assoc..

[24] Messina J, Campbell S, Morris R, Eyles E, Sanders C. A narrative systematic review of factors affecting diabetes prevention in primary care settings. PLoS One. 2017;12(5):e0177699. 10.1371/journal.pone.0177699 PMC5439678 28531197

[25] Vanstone M, Rewegan A, Brundisini F, Giacomini M, Kandasamy S, Dejean D (2017). Diet modification challenges faced by marginalized and nonmarginalized adults with type 2 diabetes: a systematic review and qualitative meta-synthesis. Chronic Illn.

[26] Wright K, Golder S, Lewis-Light K (2015). What value is the CINAHL database when searching for systematic reviews of qualitative studies?. Syst Rev..

[27] Salisbury L (2009). Web of science and Scopus: a comparative review of content and searching capabilities. Charlest Advis.

[28] Page MJ, Shamseer L, Altman DG, Tetzlaff J, Sampson M, Tricco AC, et al. Epidemiology and reporting characteristics of systematic reviews of biomedical research: a cross-sectional study. PLoS Med. 2016;13(5):e1002028. 10.1371/journal.pmed.1002028 PMC4878797 27218655

[29] US National Library of Medicine. MEDLINE, PubMed and PMC (PubMed Central) - How are they different?. Fact Sheet. 2016. p. 1. Available from: https://www.nlm.nih.gov/pubs/factsheets/dif_med_pub.html. [cited 2021 Mar 4]

